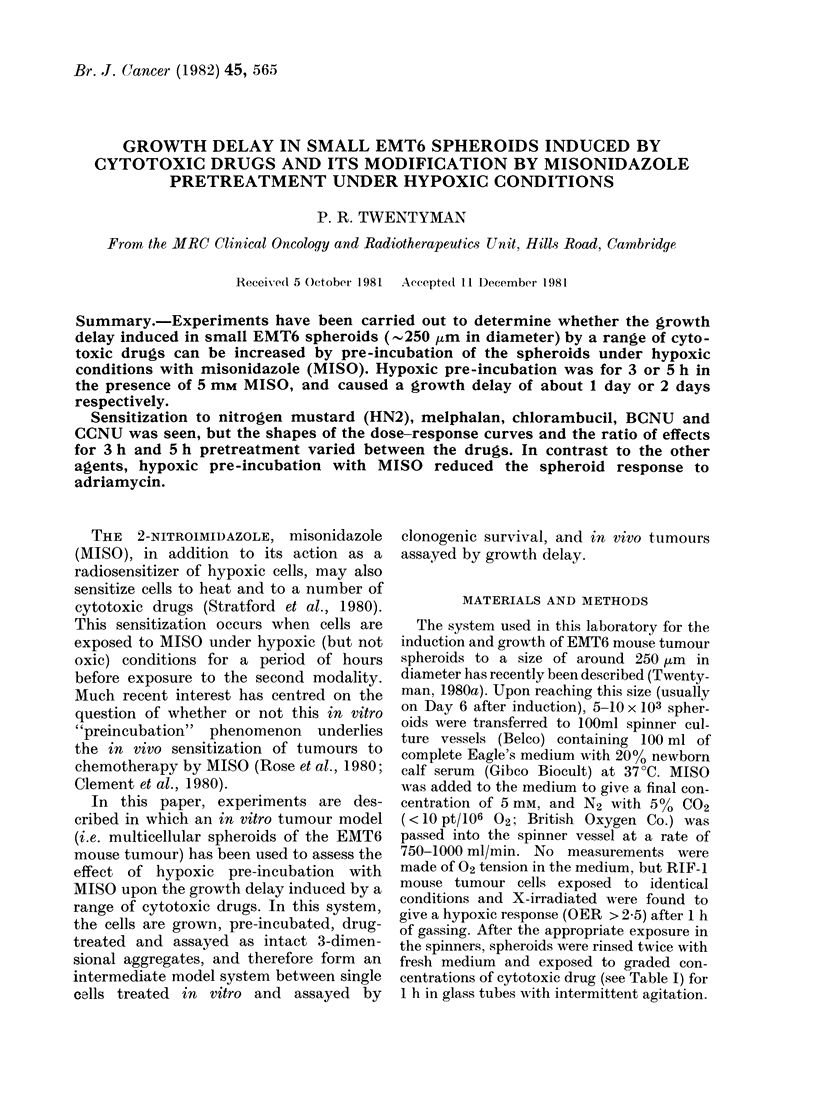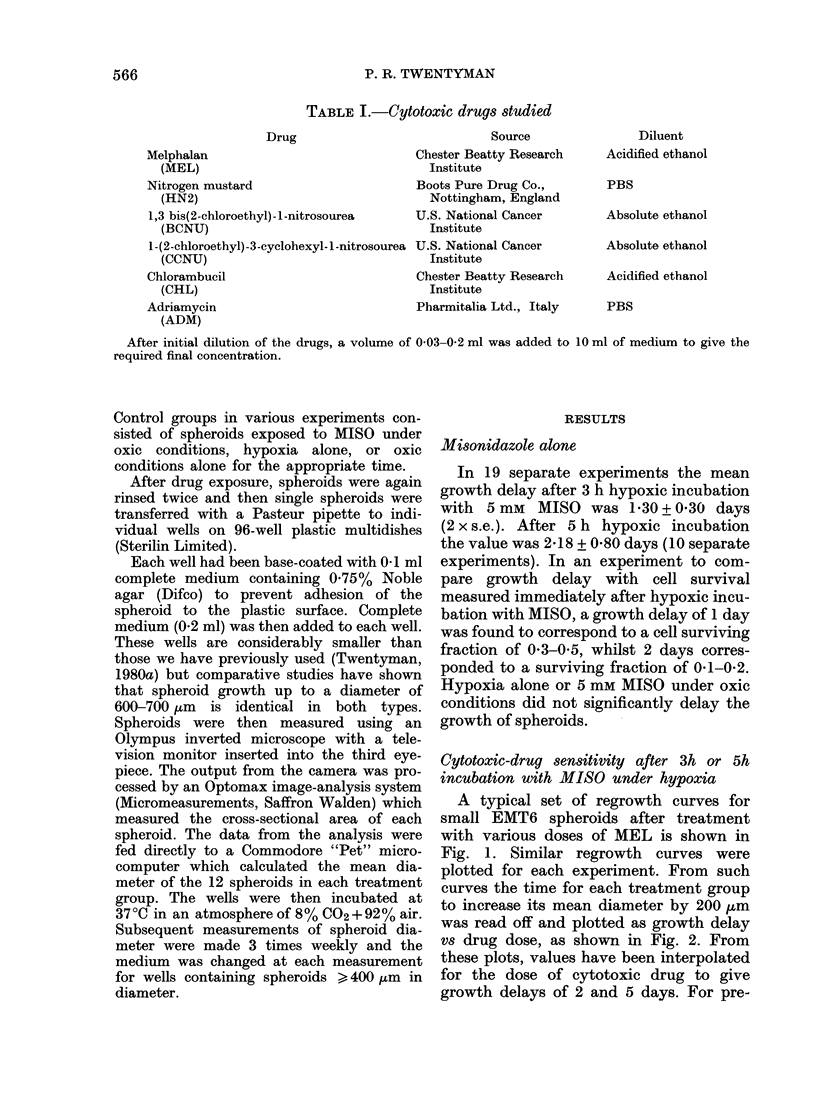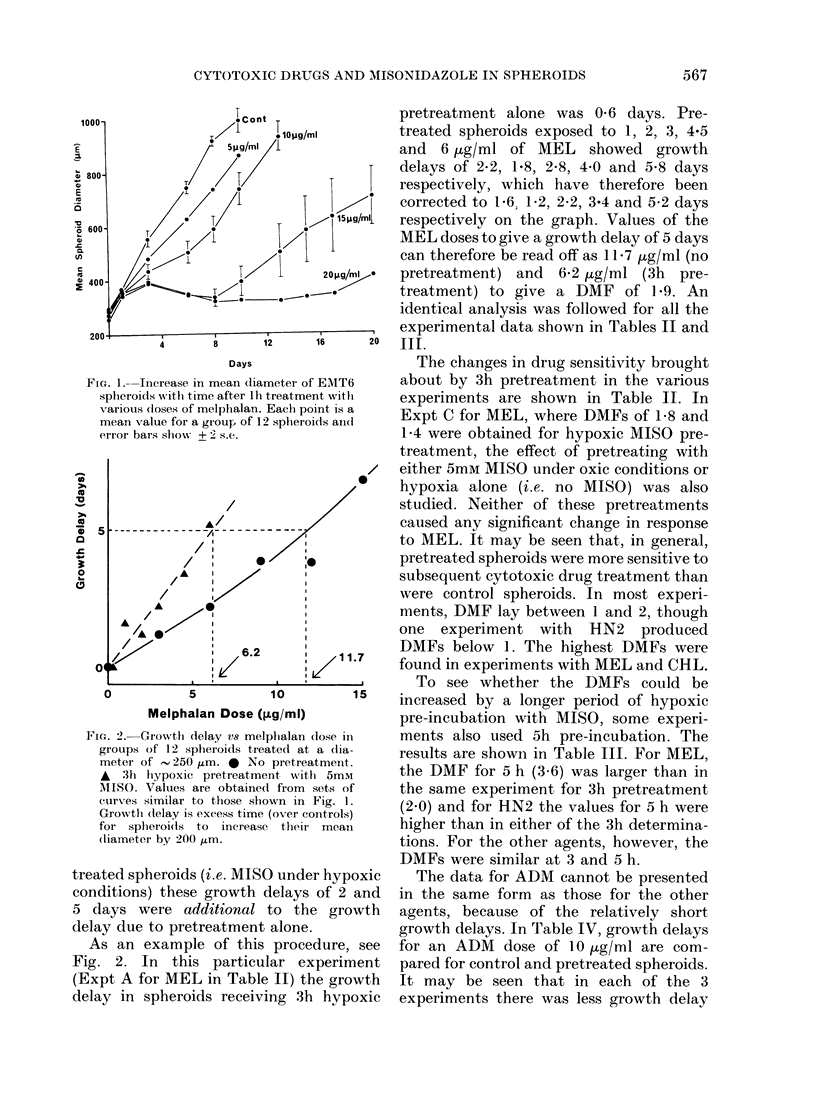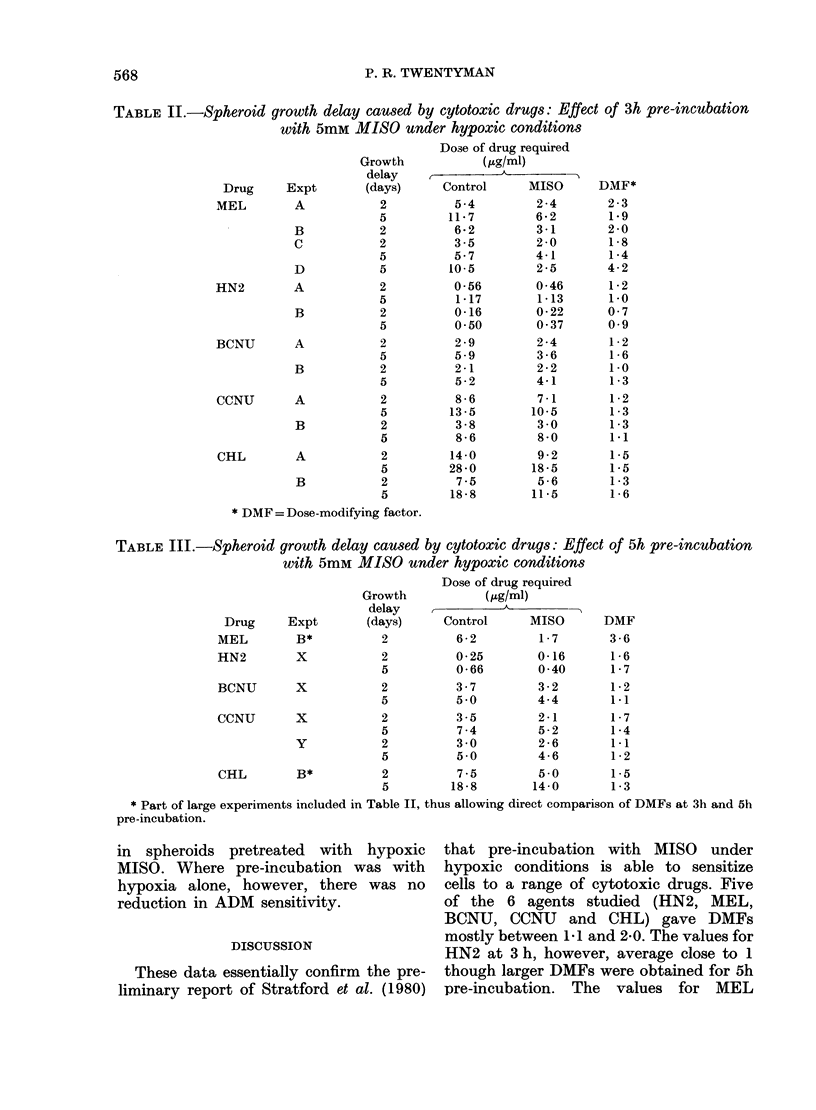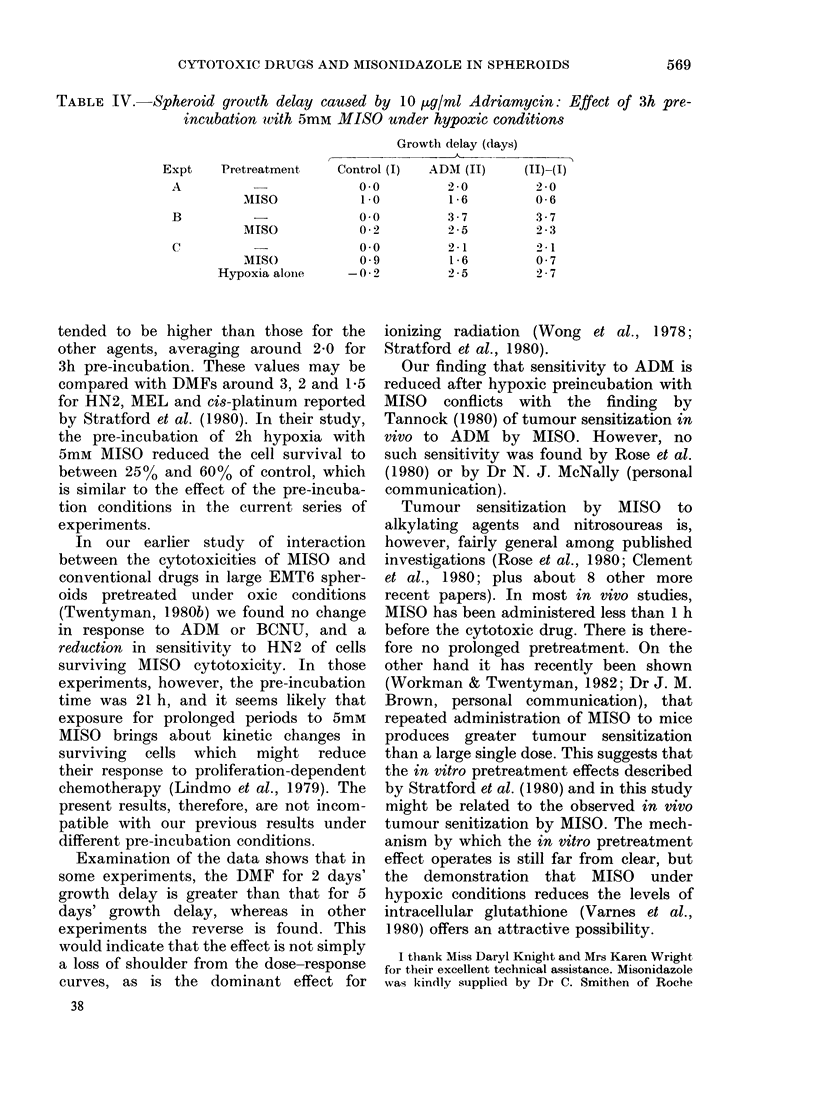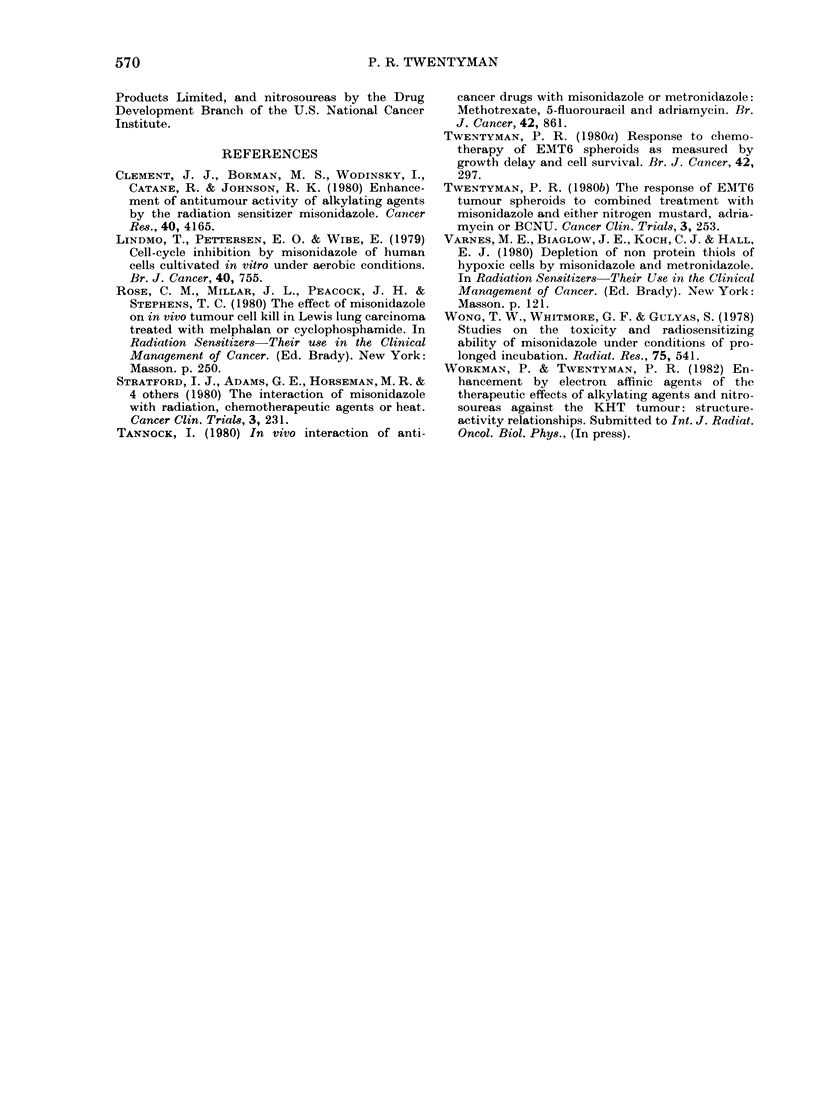# Growth delay in small EMT6 spheroids induced by cytotoxic drugs and its modification by misonidazole pretreatment under hypoxic conditions.

**DOI:** 10.1038/bjc.1982.93

**Published:** 1982-04

**Authors:** P. R. Twentyman

## Abstract

Experiments have been carried out to determine whether the growth delay induced in small EMT6 spheroids (approximately 250 micrometers in diameter) by a range of cytotoxic drugs can be increased by pre-incubation of the spheroids under hypoxic conditions with misonidazole (MISO). Hypoxic pre-incubation was for 3 or 5 h in the presence of 5 mM MISO, and caused a growth delay of about 1 day or 2 days respectively. Sensitization to nitrogen mustard (HN2), melphalan, chlorambucil, BCNU and CCNU was seen, but the shapes of the dose-response curves and the ratio of effects for 3 h and 5 h pretreatment varied between the drugs. In contrast to the other agents, hypoxic pre-incubation with MISO reduced the spheroid response to adriamycin.


*Br. J. Cancer* (1982) **45**, 565

### GROWTH DELAY IN SMALL EMT6 SPHEROIDS INDUCED BY CYTOTOXIC DRUGS AND ITS MODIFICATION BY MISONIDAZOLE PRETREATMENT UNDER HYPOXIC CONDITIONS

P. R. TWENTYMAN

*From the MRC Clinical Oncology and Radiotherapeutics Unit, Hills Road, Cambridge*

Received 5 October 1981 Accepted 11 December 1981

**Summary.—Experiments have been carried out to determine whether the growth delay induced in small EMT6 spheroids (~250 μm in diameter) by a range of cytotoxic drugs can be increased by pre-incubation of the spheroids under hypoxic conditions with misonidazole (MISO). Hypoxic pre-incubation was for 3 or 5 h in the presence of 5 mM MISO, and caused a growth delay of about 1 day or 2 days respectively.**

**Sensitization to nitrogen mustard (HN2), melphalan, chlorambucil, BCNU and CCNU was seen, but the shapes of the dose–response curves and the ratio of effects for 3 h and 5 h pretreatment varied between the drugs. In contrast to the other agents, hypoxic pre-incubation with MISO reduced the spheroid response to adriamycin.**

THE 2-NITROIMIDAZOLE, misonidazole (MISO), in addition to its action as a radiosensitizer of hypoxic cells, may also sensitize cells to heat and to a number of cytotoxic drugs (Stratford *et al.*, 1980). This sensitization occurs when cells are exposed to MISO under hypoxic (but not oxic) conditions for a period of hours before exposure to the second modality. Much recent interest has centred on the question of whether or not this *in vitro* "preincubation" phenomenon underlies the *in vivo* sensitization of tumours to chemotherapy by MISO (Rose *et al.*, 1980; Clement *et al.*, 1980).

In this paper, experiments are described in which an *in vitro* tumour model (*i.e.* multicellular spheroids of the EMT6 mouse tumour) has been used to assess the effect of hypoxic pre-incubation with MISO upon the growth delay induced by a range of cytotoxic drugs. In this system, the cells are grown, pre-incubated, drug-treated and assayed as intact 3-dimensional aggregates, and therefore form an intermediate model system between single cells treated *in vitro* and assayed by clonogenic survival, and *in vivo* tumours assayed by growth delay.

### MATERIALS AND METHODS

The system used in this laboratory for the induction and growth of EMT6 mouse tumour spheroids to a size of around 250 μm in diameter has recently been described (Twentyman, 1980*a*). Upon reaching this size (usually on Day 6 after induction), 5–10 × $10^3$ spheroids were transferred to 100ml spinner culture vessels (Belco) containing 100 ml of complete Eagle's medium with 20% newborn calf serum (Gibco Biocult) at 37°C. MISO was added to the medium to give a final concentration of 5 mM, and $N_2$ with 5% $CO_2$ (< 10 pt/$10^6$ $O_2$; British Oxygen Co.) was passed into the spinner vessel at a rate of 750–1000 ml/min. No measurements were made of $O_2$ tension in the medium, but RIF-1 mouse tumour cells exposed to identical conditions and X-irradiated were found to give a hypoxic response (OER > 2·5) after 1 h of gassing. After the appropriate exposure in the spinners, spheroids were rinsed twice with fresh medium and exposed to graded concentrations of cytotoxic drug (see Table I) for 1 h in glass tubes with intermittent agitation.

TABLE I.—*Cytotoxic drugs studied*

| Drug | Source | Diluent |
|---|---|---|
| Melphalan (MEL) | Chester Beatty Research Institute | Acidified ethanol |
| Nitrogen mustard (HN2) | Boots Pure Drug Co., Nottingham, England | PBS |
| 1,3 bis(2-chloroethyl)-1-nitrosourea (BCNU) | U.S. National Cancer Institute | Absolute ethanol |
| 1-(2-chloroethyl)-3-cyclohexyl-1-nitrosourea (CCNU) | U.S. National Cancer Institute | Absolute ethanol |
| Chlorambucil (CHL) | Chester Beatty Research Institute | Acidified ethanol |
| Adriamycin (ADM) | Pharmitalia Ltd., Italy | PBS |

After initial dilution of the drugs, a volume of 0·03–0·2 ml was added to 10 ml of medium to give the required final concentration.

Control groups in various experiments consisted of spheroids exposed to MISO under oxic conditions, hypoxia alone, or oxic conditions alone for the appropriate time.

After drug exposure, spheroids were again rinsed twice and then single spheroids were transferred with a Pasteur pipette to individual wells on 96-well plastic multidishes (Sterilin Limited).

Each well had been base-coated with 0·1 ml complete medium containing 0·75% Noble agar (Difco) to prevent adhesion of the spheroid to the plastic surface. Complete medium (0·2 ml) was then added to each well. These wells are considerably smaller than those we have previously used (Twentyman, 1980*a*) but comparative studies have shown that spheroid growth up to a diameter of 600–700 $\mu$m is identical in both types. Spheroids were then measured using an Olympus inverted microscope with a television monitor inserted into the third eyepiece. The output from the camera was processed by an Optomax image-analysis system (Micromeasurements, Saffron Walden) which measured the cross-sectional area of each spheroid. The data from the analysis were fed directly to a Commodore "Pet" microcomputer which calculated the mean diameter of the 12 spheroids in each treatment group. The wells were then incubated at 37°C in an atmosphere of 8% $CO_2$ + 92% air. Subsequent measurements of spheroid diameter were made 3 times weekly and the medium was changed at each measurement for wells containing spheroids $\geqslant$400 $\mu$m in diameter.

### RESULTS

### *Misonidazole alone*

In 19 separate experiments the mean growth delay after 3 h hypoxic incubation with 5 mM MISO was 1·30 ± 0·30 days (2 × s.e.). After 5 h hypoxic incubation the value was 2·18 ± 0·80 days (10 separate experiments). In an experiment to compare growth delay with cell survival measured immediately after hypoxic incubation with MISO, a growth delay of 1 day was found to correspond to a cell surviving fraction of 0·3–0·5, whilst 2 days corresponded to a surviving fraction of 0·1–0·2. Hypoxia alone or 5 mM MISO under oxic conditions did not significantly delay the growth of spheroids.

### *Cytotoxic-drug sensitivity after 3h or 5h incubation with MISO under hypoxia*

A typical set of regrowth curves for small EMT6 spheroids after treatment with various doses of MEL is shown in Fig. 1. Similar regrowth curves were plotted for each experiment. From such curves the time for each treatment group to increase its mean diameter by 200 $\mu$m was read off and plotted as growth delay *vs* drug dose, as shown in Fig. 2. From these plots, values have been interpolated for the dose of cytotoxic drug to give growth delays of 2 and 5 days. For pre-

FIG. 1.—Increase in mean diameter of EMT6 spheroids with time after 1h treatment with various doses of melphalan. Each point is a mean value for a group of 12 spheroids and error bars show ± 2 s.e.

FIG. 2.—Growth delay *vs* melphalan dose in groups of 12 spheroids treated at a diameter of ~250 µm. ● No pretreatment. ▲ 3h hypoxic pretreatment with 5mM MISO. Values are obtained from sets of curves similar to those shown in Fig. 1. Growth delay is excess time (over controls) for spheroids to increase their mean diameter by 200 µm.

treated spheroids (*i.e.* MISO under hypoxic conditions) these growth delays of 2 and 5 days were *additional* to the growth delay due to pretreatment alone.

As an example of this procedure, see Fig. 2. In this particular experiment (Expt A for MEL in Table II) the growth delay in spheroids receiving 3h hypoxic pretreatment alone was 0·6 days. Pretreated spheroids exposed to 1, 2, 3, 4·5 and 6 µg/ml of MEL showed growth delays of 2·2, 1·8, 2·8, 4·0 and 5·8 days respectively, which have therefore been corrected to 1·6, 1·2, 2·2, 3·4 and 5·2 days respectively on the graph. Values of the MEL doses to give a growth delay of 5 days can therefore be read off as 11·7 µg/ml (no pretreatment) and 6·2 µg/ml (3h pretreatment) to give a DMF of 1·9. An identical analysis was followed for all the experimental data shown in Tables II and III.

The changes in drug sensitivity brought about by 3h pretreatment in the various experiments are shown in Table II. In Expt C for MEL, where DMFs of 1·8 and 1·4 were obtained for hypoxic MISO pretreatment, the effect of pretreating with either 5mM MISO under oxic conditions or hypoxia alone (*i.e.* no MISO) was also studied. Neither of these pretreatments caused any significant change in response to MEL. It may be seen that, in general, pretreated spheroids were more sensitive to subsequent cytotoxic drug treatment than were control spheroids. In most experiments, DMF lay between 1 and 2, though one experiment with HN2 produced DMFs below 1. The highest DMFs were found in experiments with MEL and CHL.

To see whether the DMFs could be increased by a longer period of hypoxic pre-incubation with MISO, some experiments also used 5h pre-incubation. The results are shown in Table III. For MEL, the DMF for 5 h (3·6) was larger than in the same experiment for 3h pretreatment (2·0) and for HN2 the values for 5 h were higher than in either of the 3h determinations. For the other agents, however, the DMFs were similar at 3 and 5 h.

The data for ADM cannot be presented in the same form as those for the other agents, because of the relatively short growth delays. In Table IV, growth delays for an ADM dose of 10 µg/ml are compared for control and pretreated spheroids. It may be seen that in each of the 3 experiments there was less growth delay

TABLE II.—*Spheroid growth delay caused by cytotoxic drugs: Effect of 3h pre-incubation with 5mM MISO under hypoxic conditions*

| Drug | Expt | Growth delay (days) | Dose of drug required (μg/ml) Control | MISO | DMF* |
|---|---|---|---|---|---|
| MEL | A | 2 | 5·4 | 2·4 | 2·3 |
| | | 5 | 11·7 | 6·2 | 1·9 |
| | B | 2 | 6·2 | 3·1 | 2·0 |
| | C | 2 | 3·5 | 2·0 | 1·8 |
| | | 5 | 5·7 | 4·1 | 1·4 |
| | D | 5 | 10·5 | 2·5 | 4·2 |
| HN2 | A | 2 | 0·56 | 0·46 | 1·2 |
| | | 5 | 1·17 | 1·13 | 1·0 |
| | B | 2 | 0·16 | 0·22 | 0·7 |
| | | 5 | 0·50 | 0·37 | 0·9 |
| BCNU | A | 2 | 2·9 | 2·4 | 1·2 |
| | | 5 | 5·9 | 3·6 | 1·6 |
| | B | 2 | 2·1 | 2·2 | 1·0 |
| | | 5 | 5·2 | 4·1 | 1·3 |
| CCNU | A | 2 | 8·6 | 7·1 | 1·2 |
| | | 5 | 13·5 | 10·5 | 1·3 |
| | B | 2 | 3·8 | 3·0 | 1·3 |
| | | 5 | 8·6 | 8·0 | 1·1 |
| CHL | A | 2 | 14·0 | 9·2 | 1·5 |
| | | 5 | 28·0 | 18·5 | 1·5 |
| | B | 2 | 7·5 | 5·6 | 1·3 |
| | | 5 | 18·8 | 11·5 | 1·6 |

* DMF = Dose-modifying factor.

TABLE III.—*Spheroid growth delay caused by cytotoxic drugs: Effect of 5h pre-incubation with 5mM MISO under hypoxic conditions*

| Drug | Expt | Growth delay (days) | Dose of drug required (μg/ml) Control | MISO | DMF |
|---|---|---|---|---|---|
| MEL | B* | 2 | 6·2 | 1·7 | 3·6 |
| HN2 | X | 2 | 0·25 | 0·16 | 1·6 |
| | | 5 | 0·66 | 0·40 | 1·7 |
| BCNU | X | 2 | 3·7 | 3·2 | 1·2 |
| | | 5 | 5·0 | 4·4 | 1·1 |
| CCNU | X | 2 | 3·5 | 2·1 | 1·7 |
| | | 5 | 7·4 | 5·2 | 1·4 |
| | Y | 2 | 3·0 | 2·6 | 1·1 |
| | | 5 | 5·0 | 4·6 | 1·2 |
| CHL | B* | 2 | 7·5 | 5·0 | 1·5 |
| | | 5 | 18·8 | 14·0 | 1·3 |

* Part of large experiments included in Table II, thus allowing direct comparison of DMFs at 3h and 5h pre-incubation.

in spheroids pretreated with hypoxic MISO. Where pre-incubation was with hypoxia alone, however, there was no reduction in ADM sensitivity.

### DISCUSSION

These data essentially confirm the preliminary report of Stratford *et al.* (1980) that pre-incubation with MISO under hypoxic conditions is able to sensitize cells to a range of cytotoxic drugs. Five of the 6 agents studied (HN2, MEL, BCNU, CCNU and CHL) gave DMFs mostly between 1·1 and 2·0. The values for HN2 at 3 h, however, average close to 1 though larger DMFs were obtained for 5h pre-incubation. The values for MEL

TABLE IV.—*Spheroid growth delay caused by* 10 *μg/ml Adriamycin: Effect of* 3*h pre-incubation with* 5mM *MISO under hypoxic conditions*

| | | Growth delay (days) | | |
|---|---|---|---|---|
| Expt | Pretreatment | Control (I) | ADM (II) | (II)–(I) |
| A | — | 0·0 | 2·0 | 2·0 |
| | MISO | 1·0 | 1·6 | 0·6 |
| B | — | 0·0 | 3·7 | 3·7 |
| | MISO | 0·2 | 2·5 | 2·3 |
| C | — | 0·0 | 2·1 | 2·1 |
| | MISO | 0·9 | 1·6 | 0·7 |
| | Hypoxia alone | −0·2 | 2·5 | 2·7 |

tended to be higher than those for the other agents, averaging around 2·0 for 3h pre-incubation. These values may be compared with DMFs around 3, 2 and 1·5 for HN2, MEL and *cis*-platinum reported by Stratford *et al.* (1980). In their study, the pre-incubation of 2h hypoxia with 5mM MISO reduced the cell survival to between 25% and 60% of control, which is similar to the effect of the pre-incubation conditions in the current series of experiments.

In our earlier study of interaction between the cytotoxicities of MISO and conventional drugs in large EMT6 spheroids pretreated under oxic conditions (Twentyman, 1980*b*) we found no change in response to ADM or BCNU, and a *reduction* in sensitivity to HN2 of cells surviving MISO cytotoxicity. In those experiments, however, the pre-incubation time was 21 h, and it seems likely that exposure for prolonged periods to 5mM MISO brings about kinetic changes in surviving cells which might reduce their response to proliferation-dependent chemotherapy (Lindmo *et al.*, 1979). The present results, therefore, are not incompatible with our previous results under different pre-incubation conditions.

Examination of the data shows that in some experiments, the DMF for 2 days' growth delay is greater than that for 5 days' growth delay, whereas in other experiments the reverse is found. This would indicate that the effect is not simply a loss of shoulder from the dose–response curves, as is the dominant effect for ionizing radiation (Wong *et al.*, 1978; Stratford *et al.*, 1980).

Our finding that sensitivity to ADM is reduced after hypoxic preincubation with MISO conflicts with the finding by Tannock (1980) of tumour sensitization *in vivo* to ADM by MISO. However, no such sensitivity was found by Rose *et al.* (1980) or by Dr N. J. McNally (personal communication).

Tumour sensitization by MISO to alkylating agents and nitrosoureas is, however, fairly general among published investigations (Rose *et al.*, 1980; Clement *et al.*, 1980; plus about 8 other more recent papers). In most *in vivo* studies, MISO has been administered less than 1 h before the cytotoxic drug. There is therefore no prolonged pretreatment. On the other hand it has recently been shown (Workman & Twentyman, 1982; Dr J. M. Brown, personal communication), that repeated administration of MISO to mice produces greater tumour sensitization than a large single dose. This suggests that the *in vitro* pretreatment effects described by Stratford *et al.* (1980) and in this study might be related to the observed *in vivo* tumour senitization by MISO. The mechanism by which the *in vitro* pretreatment effect operates is still far from clear, but the demonstration that MISO under hypoxic conditions reduces the levels of intracellular glutathione (Varnes *et al.*, 1980) offers an attractive possibility.

I thank Miss Daryl Knight and Mrs Karen Wright for their excellent technical assistance. Misonidazole was kindly supplied by Dr C. Smithen of Roche

Products Limited, and nitrosoureas by the Drug Development Branch of the U.S. National Cancer Institute.

### REFERENCES

CLEMENT, J. J., BORMAN, M. S., WODINSKY, I., CATANE, R. & JOHNSON, R. K. (1980) Enhancement of antitumour activity of alkylating agents by the radiation sensitizer misonidazole. *Cancer Res.*, **40,** 4165.

LINDMO, T., PETTERSEN, E. O. & WIBE, E. (1979) Cell-cycle inhibition by misonidazole of human cells cultivated *in vitro* under aerobic conditions. *Br. J. Cancer*, **40,** 755.

ROSE, C. M., MILLAR, J. L., PEACOCK, J. H. & STEPHENS, T. C. (1980) The effect of misonidazole on *in vivo* tumour cell kill in Lewis lung carcinoma treated with melphalan or cyclophosphamide. In *Radiation Sensitizers—Their use in the Clinical Management of Cancer.* (Ed. Brady). New York: Masson. p. 250.

STRATFORD, I. J., ADAMS, G. E., HORSEMAN, M. R. & 4 others (1980) The interaction of misonidazole with radiation, chemotherapeutic agents or heat. *Cancer Clin. Trials*, **3,** 231.

TANNOCK, I. (1980) *In vivo* interaction of anticancer drugs with misonidazole or metronidazole: Methotrexate, 5-fluorouracil and adriamycin. *Br. J. Cancer*, **42,** 861.

TWENTYMAN, P. R. (1980*a*) Response to chemotherapy of EMT6 spheroids as measured by growth delay and cell survival. *Br. J. Cancer*, **42,** 297.

TWENTYMAN, P. R. (1980*b*) The response of EMT6 tumour spheroids to combined treatment with misonidazole and either nitrogen mustard, adriamycin or BCNU. *Cancer Clin. Trials*, **3,** 253.

VARNES, M. E., BIAGLOW, J. E., KOCH, C. J. & HALL, E. J. (1980) Depletion of non protein thiols of hypoxic cells by misonidazole and metronidazole. In *Radiation Sensitizers—Their Use in the Clinical Management of Cancer.* (Ed. Brady). New York: Masson. p. 121.

WONG, T. W., WHITMORE, G. F. & GULYAS, S. (1978) Studies on the toxicity and radiosensitizing ability of misonidazole under conditions of prolonged incubation. *Radiat. Res.*, **75,** 541.

WORKMAN, P. & TWENTYMAN, P. R. (1982) Enhancement by electron affinic agents of the therapeutic effects of alkylating agents and nitrosoureas against the KHT tumour: structure-activity relationships. Submitted to *Int. J. Radiat. Oncol. Biol. Phys.*, (In press).